# Synergistic activity of gold nanoparticles with amphotericin B on persister cells of *Candida tropicalis* biofilms

**DOI:** 10.1186/s12951-024-02415-6

**Published:** 2024-05-16

**Authors:** M. A. Dasilva, K. F. Crespo Andrada, M. Maldonado Torales, I. Manrrique Hughes, P. Pez, J. C. García-Martínez, María Gabriela Paraje

**Affiliations:** 1 Instituto Multidisciplinario de Biología Vegetal (IMBIV), Consejo Nacional de Investigaciones Científicas y Técnicas (CONICET), Córdoba, Argentina; 2 Cátedra de Microbiología, Facultad de Ciencias Exactas, Físicas y Naturales, Universidad Nacional de Córdoba, Córdoba, X5000HUA Argentina; 3 Departamento de Ciencias Farmacéuticas, Facultad de Ciencias Químicas, Universidad Nacional de Córdoba, Córdoba, Argentina; 4 Facultad de Farmacia de Albacete, Centro Regional de Investigaciones Biomédicas, Universidad de Castilla-La Mancha, Ciudad Real, España

**Keywords:** Antibiofilm activity, Gold nanoparticles, Amphotericin B, Synergy, *Candida tropicalis*, Sessile cells, Persister cells

## Abstract

**Aim:**

The antifungal activity was studied on sessile and persister cells (PCs) of *Candida tropicalis* biofilms of gold nanoparticles (AuNPs) stabilized with cetyltrimethylammonium bromide (CTAB-AuNPs) and those conjugated with cysteine, in combination with Amphotericin B (AmB).

**Materials/methods:**

The PC model was used and synergistic activity was tested by the checkerboard assay. Biofilms were studied by crystal violet and scanning electron microscopy.

**Results/Conclusions:**

After the combination of both AuNPs and AmB the biofilm biomass was reduced, with significant differences in architecture being observed with a reduced biofilm matrix. In addition, the CTAB-AuNPs-AmB combination significantly reduced PCs. Understanding how these AuNPs aid in the fight against biofilms and the development of new approaches to eradicate PCs has relevance for chronic infection treatment.

## Introduction

*Candida tropicalis* has been included as an emergent pathogen in the “high group” of the World Health Organization Antimicrobial Resistance Division fungal priority pathogens list [1]. An important virulence factor crucial to the development of chronic infections is its ability to form biofilms on host cells, with 90% of *Candida* infections being related to biofilms [2–4]. Biphasic killing implies the existence of a small fraction of a highly tolerant cell population within the biofilms, called persister cells (PCs) [5–7]. PCs are phenotypic variants of a wild type and are transiently refractory to the killing, without having acquired resistance through genetic modification. These are associated with recurrent or chronic infections [8, 9].

To date, only a few antifungal drugs are partially effective against *Candida* biofilm-associated infections, of which the gold standard in *Candida* infections is Amphotericin B (AmB) [10–12]. However, based on the Food and Drug Administration Adverse Event Reporting System database, the use of AmB is often limited by the fact that it is associated with nephrotoxicity, hepatotoxicity, hematotoxicity, and hypersensitivity reactions [13, 14]. New therapies, such as the use of novel compounds alone or combined with a first-line antifungal agent, could provide an effective solution for *Candida* biofilm infections, thereby improving the efficacy and reducing side effects [15–17]. A promising direction for future research is using metallic nanoparticles, such as gold nanoparticles (AuNPs), as well as their combination with other antifungal agents [18–21].

The present study was performed with the aim of determining the antibiofilm effect of AuNPs stabilized with cetyltrimethylammonium bromide (CTAB-AuNPs) or conjugated with cysteine (cys-AuNPs), in combination with AmB, on sessile cells and PCs of *C. tropicalis* biofilms. To carry this out, a PC experimental model previously described was used [7]. The synergistic activity was tested by the checkerboard titration method. Scanning electron microscopy (SEM) was used to enable a more detailed analysis of the antibiofilm effect on sessile cells and PCs, as well as to determine their spatial organization. To our knowledge, this is the first study that has attempted to correlate the PCs of *Candida* biofilm reduction with the action of AuNPs combined with AmB and to investigate the consequences on the surface topography and the three-dimensional architecture of the biofilms. Our improved understanding of how these AuNPs aid in the fight against biofilm-associated candidiasis and the development of new approaches to eradicate PC biofilms could have great clinical relevance in the treatment of mycoses, which has seen an increasing number of at-risk patients.

## Materials and methods

### Fungal strain and antifungal agents

*C. tropicalis* NCPF 3111 (National Collection of Pathogenic Fungi, Bristol, UK) a reference strain was used. Standardized cellular suspensions (1 × 10^7^ ml^− 1^) in Sabouraud Dextrose Broth (SDB; Difco, MI, USA) were obtained from an overnight culture at 37 °C. Stock cultures at − 80 °C were grown on SDB with glycerol (15% v/v) (Anedra, Buenos Aires, Argentina) and subcultured twice from frozen stocks before each experiment onto Sabouraud Dextrose Agar (SDA; Difco) at 37 °C. The visibility and purity of the colonies were checked on SDA plates [7, 16, 19, 22, 23].

The entire procedure employed in synthesizing and characterizing the AuNPs stabilized with cetyltrimethylammonium bromide and those conjugated with cysteine was carried out according to methods described in previous investigations [19, 24–26]. The AuNPs obtained had an aspect ratio close to 12.4 ± 0.3 and 10.8 ± 0.4 nm for CTAB-AuNPs and cys-AuNPs, respectively, with low polydispersity and spherical forms. Zeta scores of + 57.5 ± 4.3 and − 40.93 ± 0.9 mV and plasmon bands centered at 482 and 514 nm were reported for CTAB-AuNPs and cys-AuNPs, respectively [19].

AmB (80% pure HPLC grade, Sigma-Aldrich, St. Louis, MO, USA) was used as an antifungal reference agent dissolved with 1% (v/v) of dimethyl sulfoxide (DMSO Merck, Darmstadt, Germany). Previously, we reported the minimum inhibitory concentration (MIC) and minimum fungicidal concentration (MFC) endpoints for AuNPs, cys-AuNPs, and AmB on planktonic cells of *C. tropicalis* (Table 1) [7, 19, 27].

**Table 1:** Minimum inhibitory concentration (MIC) and minimum fungicidal concentration (MFC) on planktonic cells, and minimal biofilm inhibitory concentration (MBIC) and minimal biofilm eradication concentration (MBEC) for gold nanoparticles (AuNPs) and Amphotericin B (AmB) acting on sessile cells on *Candida tropicalis*

	Planktonic cells				Sessile cells			
	MIC	MFC	MFC/MIC	INTPN	MBIC	MBEC	MBEC/MBIC	-fold higher MFC
AmB	0.25	0.25	1	Fungicidal	200	200	1	800
CTAB-AuNPs	0.02	0.02	1	Fungicidal	0.31	0.63	2	31.50
cys-AuNPs	0.04	0.04	1	Fungicidal	0.63	0.63	1	15.75

**Abbreviations**: Amphotericin B (AmB), cetyltrimethylammonium bromide gold nanoparticles (CTAB-AuNPs) and conjugated with cysteine (cys-AuNPs), minimum inhibitory concentration (MIC), fractional inhibitory concentration index (FICi), interpretation (INTPN)

**Note**: FICi scores are the average of a minimum of two independent replicates

### Mature biofilm formation and PC quantification assay

**Fig. 1:**
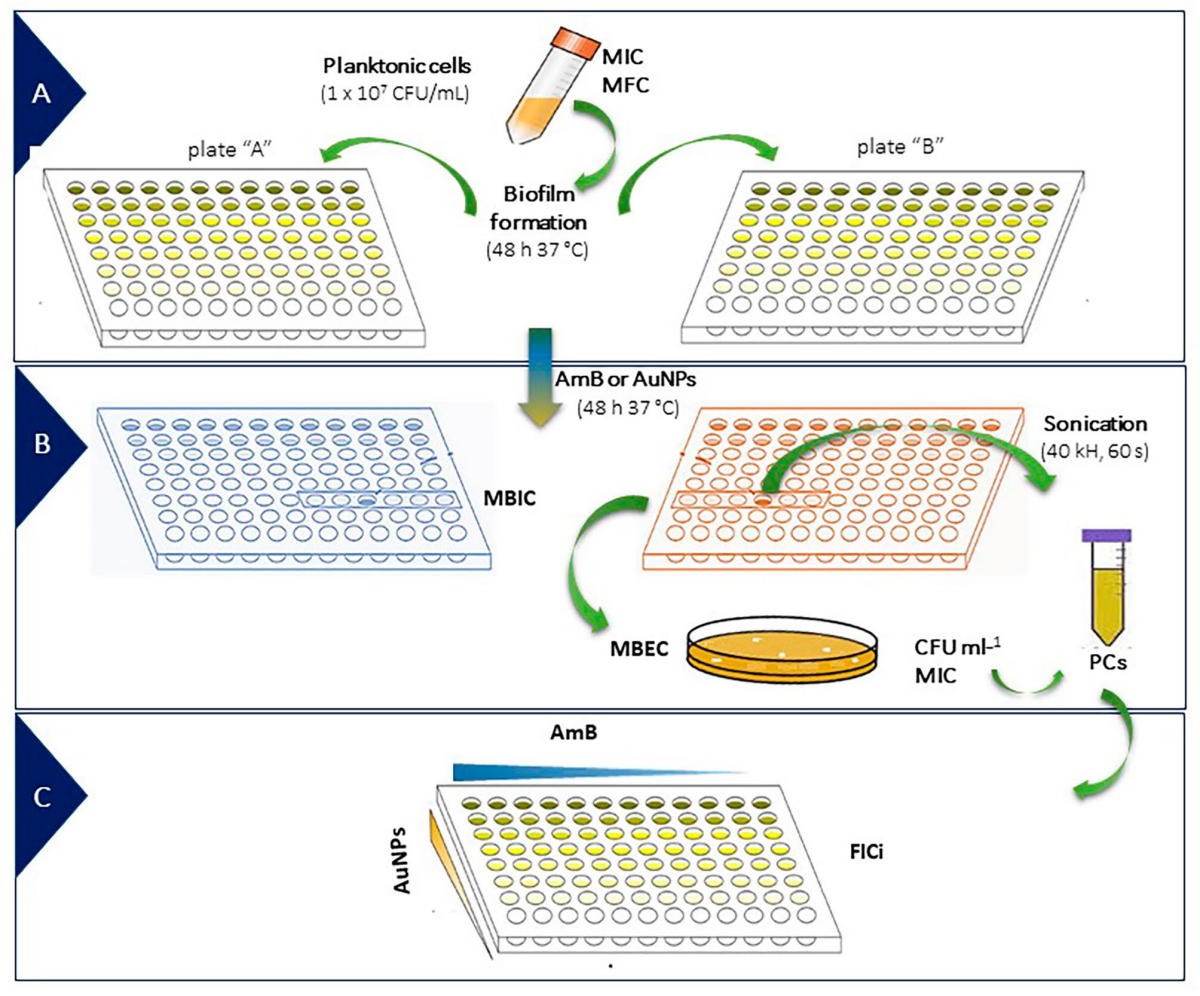
Experimental model for the study of synergistic antifungal activity of gold nanoparticles with Amphotericin B-tolerant persister cells (PCs) on *Candida tropicalis.* (**A**) *C. tropicalis* mature biofilm formation, (**B**) susceptibility endpoint biofilm parameter determination and PC quantification assay, (**C**) checkerboard microdilution assay used to evaluate the synergistic antifungal effect as a consequence of interaction. Abbreviations: Amphotericin B (AmB), gold nanoparticles (AuNPs), minimum inhibitory concentration (MIC), minimum fungicidal concentration (MFC), biofilm biomass unit (BBU), persister cells (PCs), colony-forming units per milliliter (CFU ml^− 1^), minimal biofilm inhibitory concentration (MBIC), minimal biofilm eradication concentration (MBEC), fractional inhibitory concentration index (FICi).

For mature biofilm formation, we used Roswell Park Memorial Institute (RPMI) 1640 medium (Sigma-Aldrich Co, St. Louis, MO, USA) with 2% D-glucose and glutamine, and pH was buffered with 0.165 M morpholino propanesulphonic acid (Sigma-Aldrich Co, St. Louis, MO, USA) adjusted to pH 7.0 ± 0.1. Previously was treated with fetal bovine serum (Sigma-Aldrich Co. St. Louis, MO, USA) and biofilms were grown in two parallel flat-bottom 96-multiwell plates (Fig. 1A, plate “A” and “B”) for 48 h at 37 °C (Greiner Bio-One, Nurtingen, Germany). Multi-well plates were incubated at 37 °C in static conditions to allow biofilms to form. Biofilm biomass was quantified by crystal violet staining (CV, 1% w/v, Anedra Tigre, Argentina), suspended in 70:30 ethanol/acetone solution, and quantified at 595 nm by spectrophotometry (Infinite F50 Model, Tecan, AUS). Growth control corresponding to 48 h-old mature biofilms was included in all experiments (untreated biofilms), and the optical density (OD) from negative control (without yeast cells) wells was subtracted from all tested wells. The biofilm biomass unit (BBU) was previously defined as 0.1 OD_595_ equal to 1 BBU [7, 16, 19, 22, 23, 28]. Mature biofilms were exposed to AmB or each AuNP at 37 °C for another 48 h (Fig. 1B). The supernatant was eliminated, and in plate “A” the minimal biofilm inhibitory concentration (MBIC) as BBU was determined.

The supernatant of plate “B” was discarded and the wells were washed with phosphate-buffered saline (PBS, Sigma Aldrich Co., St. Louis, MO, USA) at pH 7.1 ± 0.2. The adhered cells were sonicated at 40 kHz for 60 s for viable sessile cell recuperation. Serial dilutions (1:10) were made in PBS and each dilution was pipetted out (100 µl) and spread on SDA plates to determine the minimal biofilm eradication concentration (MBEC) by colony-forming units per milliliter (CFU ml^− 1^) [7, 29]. As previously reported by several researchers and official agencies, the concentrations required to eradicate mature biofilms did not match the MIC and MFC, making it necessary to establish new susceptibility endpoint parameters specific to the biofilms. Some researchers have defined the minimum concentration as that being capable of causing ≥ 80% biomass reduction (BBU) of a pre-formed mature biofilm, such as the MFC at the planktonic level. MBIC is the lowest concentration at which there is no time-dependent increase in the mean number of viable sessile cells when an early exposure time is compared with a later exposure time [30, 31].

For PC determination, dose-dependent killing was expressed as the BBU vs. different concentrations (MBEC/6, MBEC/4, MBEC/2, MBEC, MBEC*2, MBEC*4) on mature biofilms (data not shown) [6, 19, 32, 33]. The MIC determination was analyzed routinely for PC phenotype verification [6, 19].

### Checkerboard microdilution assay

The checkerboard method, considered to be the reference method, was used to evaluate the synergistic antifungal effect as a consequence of AuNPs/AmB interaction (Fig. 1C). This is a broth micro-dilution technique and was used in 96-well cell culture plates in which each row and each column contained twofold serial dilutions of AmB and AuNPs were added simultaneously in a 1:1 ratio, thereby providing a unique combination of the two agents in each well. Concentrations of around and below the MBIC were used (MBIC/6, MBIC/4, MBIC/2, MBIC, MBIC*2, MBIC*4) [34].

The fractional inhibitory concentration (FIC) is a widely accepted means of measuring interactions by calculating the fractional inhibitory concentration index (FICi) using the following equation:

FICi = (MBIC in combination/MBIC AuNPs alone) + (MBIC in combination/MBIC AmB alone).

The interaction was scored as: ≤ 0.5 = synergistic; > 0.5 to ≤ 1.0 = additive; >1.0 and ≤ 4.0 = indifferent; > 4.0 = antagonistic [35, 36].

### Theoretical response surface modeling

In this study, the Bliss independence model was chosen as a theoreticalapproach to compare the effects of AuNPs in combination with AmB. The data interpretation was made by response surface analysis, enabling a theoretical response surface of the interaction to be calculated. Previously, experimental results generated with the microplates were expressed as the percentage of growth compared to the growth control. All calculations were performed using Combenefit software (http://sourceforge.net/projects/combenefit/) [37, 38]. The synergy distribution in this study was evaluated using the SUM-SYN-ANT metric, offering a quantitative assessment of the total sum of statistically significant synergistic and antagonistic interactions for each checkerboard analysis [35, 36]. The SUM-SYN-ANT parameter played a crucial role in summarizing the overall interaction surface for all combinations studied, and its derivation was based on the Bliss independence model.

### Ultrastructural analysis of biofilm topography and architecture by SEM

The yeast morphology and visualization of sessile cells and PCs, together with the topography and architecture of biofilms, were monitored by SEM (Sigma 300VP; Carl ZEISS, Oberkochen, Germany) and performed onto 5 mm-side silicon stubs placed into a 24 wells-microtiter plate (Greiner Bio-One, Germany) following the same procedure described above. Afterward, samples were fixed, dried by the critical point drying method, and coated with carbon (graphite). Micrographs were obtained from 10 randomly selected positions (number of fields of view) using a magnification of 500x to 5000x. Then, the histogram of sessile cell size distribution was determined based on the length (µm) of yeast cells after different treatments. The mean, median, and mode were calculated from the histogram analysis of cell size distribution [19, 39].

### Statistical analysis

The experiments were carried out using groups of three and performed three times. The averages and the numerical data are presented as means ± SD. Data were analyzed by using ANOVA followed by the Student-Newman-Keuls test for multiple comparisons. * *p* < 0.01 was considered significant for comparisons with untreated samples. ^#^Denotes differences considered significant at ^#^*p* < 0.01 for comparisons between CTAB-AuNPs and cys-AuNPs. A graphical statistical analysis was performed using GraphPad Prism 6.0 (GraphPad Software, San Diego, CA, USA) and Microsoft Excel (Microsoft Corp., Redmond, WA, USA).

## Results

The synergistic antifungal activity of CTAB-AuNPs or cys-AuNPs with AmB against *C. tropicalis* was evaluated. Previously, we reported for planktonic cells that the MIC and MFC values for AmB were the same (0.25 µg ml^− 1^ − 2.7 10^− 7^ mM), and that CTAB-AuNPs (0.02 mM) showed greater antifungal activity than cys-AuNPs (0.04 mM) with MFC/MIC = 1. Regarding the antibiofilm activity, the MBIC values were 200 µg ml^− 1^ (2.16 10^− 4 |^mM) for AmB and 0.31 and 0.63 mM for CTAB-AuNPs and cys-AuNPs, respectively [7, 19].

Whereas MBIC determination is relevant for combination studies, such as MIC in planktonic cells, MBEC determination is most relevant for agents that can kill sessile cells and may be used to design antibiofilm therapy. To assess biofilm eradication (MBEC), biofilms were grown for 24 h, after which the AuNPs were added and the biofilms were additionally incubated for another 24 h in the presence of each agent. The MBEC value found (200 µg ml^− 1^ for AmB; MBEC/MBIC = 1) was 800-fold higher than the corresponding MFC. For CTAB-AuNPs, the MBEC was 0.63 mM (MBEC/MBIC = 2), with the value being 31.5-fold higher than the corresponding MFC. The same value of MBEC was obtained for cys-AuNPs, but this was 15.75-fold higher than the corresponding MFC (Table 1).

**Fig. 2:**
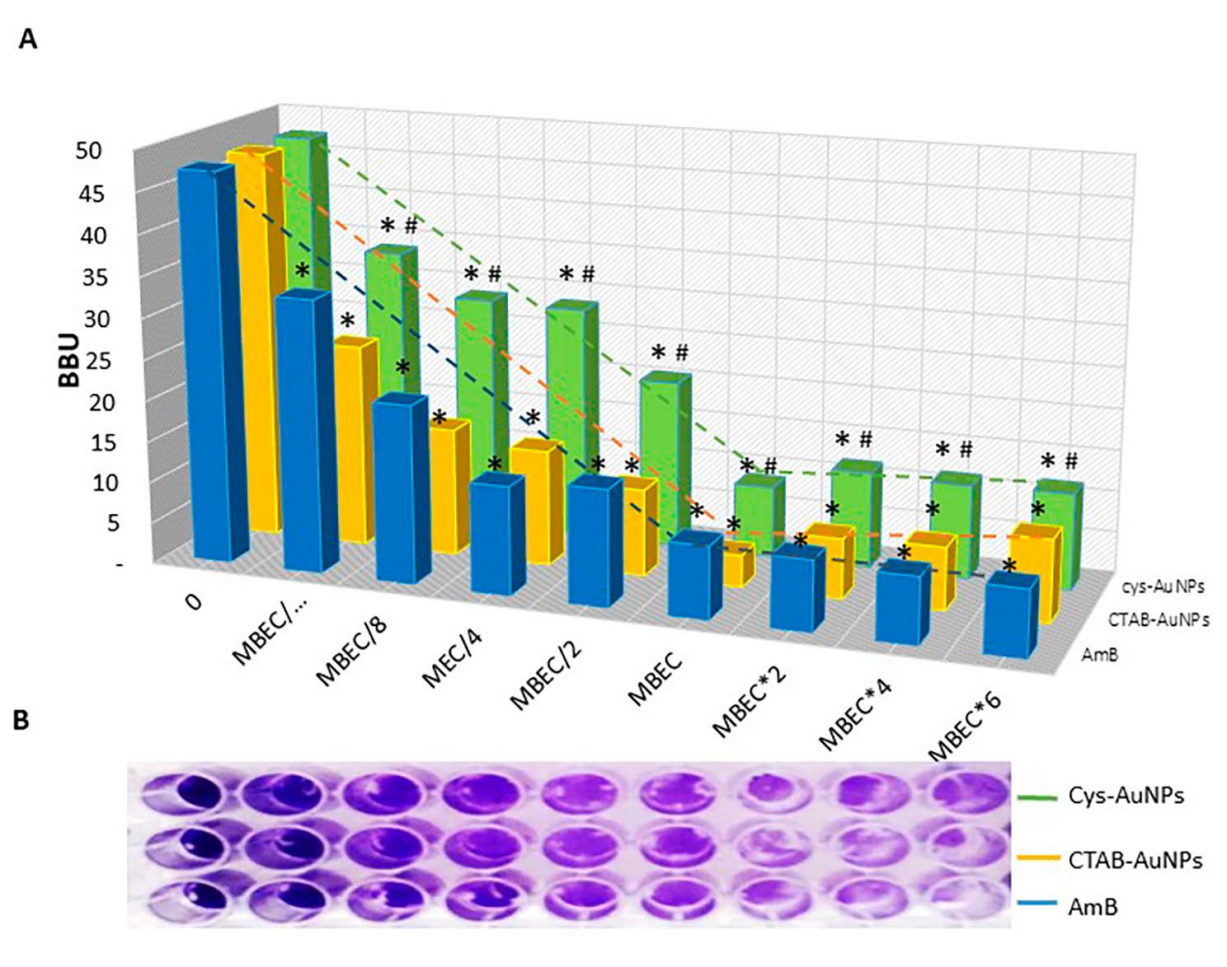
Quantification of antifungal-tolerant persister cell populations (PCs). (**A**) Dose-dependent killing with a biphasic killing pattern characteristic of a PC (% survival- line) population in response to 48 h-old *Candida tropicalis* biofilm biomass (BBU-bar) treated with Amphotericin B (AmB), cetyltrimethylammonium bromide gold nanoparticles (CTAB-AuNPs) or conjugated with cysteine (cys-AuNPs). (**B**) Biofilm quantified by crystal violet staining (top plate view). Error bars represent the standard deviations of the means of three independent experiments performed in triplicate. * *p* < 0.01 was considered significant for comparisons with untreated samples. ^#^Denotes differences considered significant at ^#^*p* < 0.01 for comparisons between CTAB-AuNPs and cys-AuNPs.

Dose-dependent killing has been shown to be an effective and straightforward method for detecting and isolating PCs. We previously reported that the percentage of surviving sessile cells AmB was 0.39%, a value indicating the presence of AmB-tolerant PCs in the *C. tropicalis* biofilms [7]. Figure 2A shows that the biphasic killing pattern revealed the presence of PCs corresponding to 1.6% for CTAB-AuNPs and 1.9% for cys-AuNPs at MBEC, respectively. The fraction of surviving cells (% survival line) was calculated by viable colony-forming units, and the PC phenotype was confirmed by evaluating the MIC in each assay, with the latter remaining unchanged. The biofilm biomass units (BBU-bar) were quantified by CV staining, and the microtiter plate assays for different MBECs are shown in Fig. 2B.

**Fig. 3:**
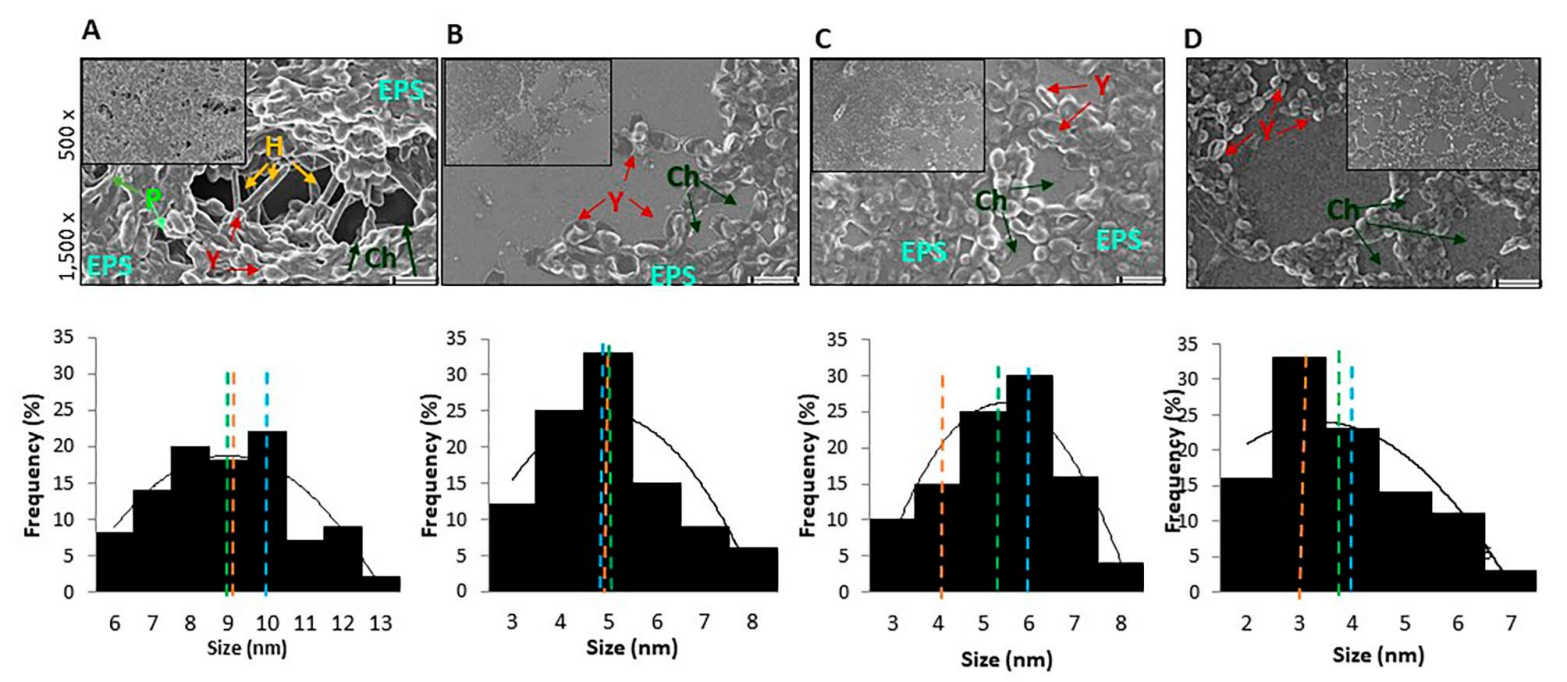
Scanning electron microscopy images and the size distribution histogram analysis of biofilm of *C. tropicalis* treated with gold nanoparticles (AgNPs) or Amphotericin B (AmB). Upper panel: Mature biofilms without treatment have a larger area of distribution and remain unaltered (**A**). After different treatments with cetyltrimethylammonium bromide gold nanoparticles (**B** - CTAB-AuNPs) conjugated with cysteine (**C** -cys-AuNPs) and AmB (**D**) at minimal biofilm eradication concentration (MBEC), a noticeable reduction is observed in the biofilm formation of the AgNPs-treated biofilms. The first panel is at 1500×, and 500× magnification is shown in the inset. True hyphae (H), yeast (Y), extracellular matrix (EPS), micropores, and internal water channels (P and Ch) are indicated by arrows. Scale bar, 10 μm. Lower panel: The mean (green), median (orange), and mode (light blue) are shown in the histogram analysis of the sessile cell size distribution for each treatment

The mature biofilm was observed using SEM in order to determine the morphological structural changes in biofilms, sessile cells, and PCs after the different treatments. The untreated mature biofilm revealed a fully covered compact 3D architecture, with abundant fungal cells with true hyphae, pseudohyphae, and yeast, embedded within a self-produced protective extracellular matrix (ECM) that revealed internal water channels and micropores. At the same time, the dense structure of the biofilms became dispersed after each AuNP was applied alone or with AmB. Pseudohyphae and true hyphae were mostly absent from these biofilms, the EPS was reduced or eliminated, and less cell clustering was observed (Fig. 3). The histogram analysis of cell size distribution was carried out based on the length of yeast cells after the different treatments. The AuNPs caused alterations in the shape and size of individual cells. CTAB-AuNPs showed the predominance of yeast’s relatively small cells, with the average (green) sessile cell size estimated to be within 5.00 ± 0.25 μm. With the addition of cysteine during AuNP biosynthesis, only insignificant differences were noted in the morphology (5.39 ± 0.35 μm). When the mature biofilms were treated with AmB, the average sizes decreased to 3.81 ± 0.33 μm. In untreated samples that showed pseudohyphae or true hyphae, the size increased on average (mean) by 8.98 ± 0.69 μm. The median for distribution (orange) is one of the most meaningful for size analysis and is referred to as D50. This value was 9 μm for untreated biofilms, 5 and 6 μm for CTAB-AuNPs and cys-AuNPs, respectively, and 3 μm for AmB. The mode is shown in the light blue line and represents the size most commonly found in the distribution, which was 10 for untreated samples, 4 for AmB, and 5 and 6 for CTAB-AuNPs and cys-AuNPs, respectively.

**Fig. 4:**
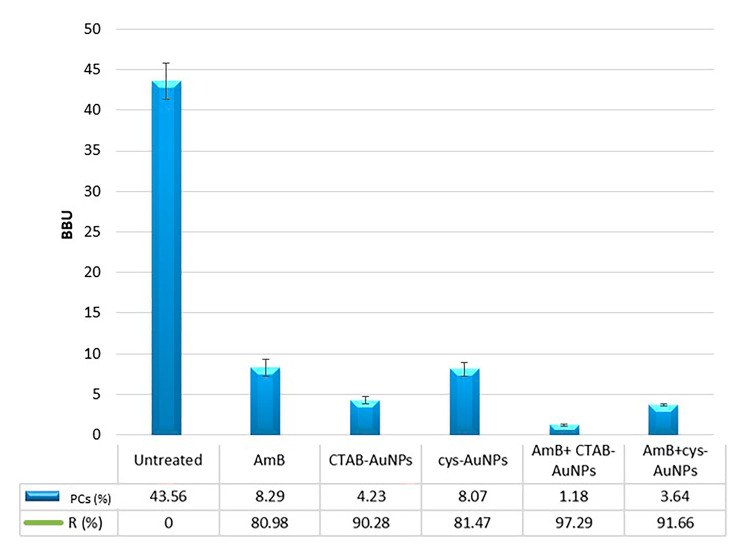
Synergic antifungal activity of AuNPs with AmB. Quantification of mature *Candida tropicalis* biofilms exposed to cetyltrimethylammonium bromide gold nanoparticles (B - CTAB-AuNPs) conjugated with cysteine (C - cys-AuNPs) and Amphotericin B (AmB) at minimal biofilm inhibitory concentration (MBIC), expressed in biofilm biomass units (BBU - bars). The fraction of surviving cells (% persister cells (PCs) was calculated in viable colony-forming units and is shown in the lower table. Inset: Fractional inhibitory concentration index (FICi) interpretations. All experiments were performed in triplicate, for three independent experiments, and the numerical data are presented as means ± standard deviation. *Denotes differences are statistically significant at *p* < 0.01 compared with untreated biofilms. ^#^Denotes differences considered significant at *p* < 0.01 for comparisons between CTAB-AuNPs and cys-AuNPs. FICi scores presented in the figures are the average of a minimum of two independent replicates

**Fig. 5:**
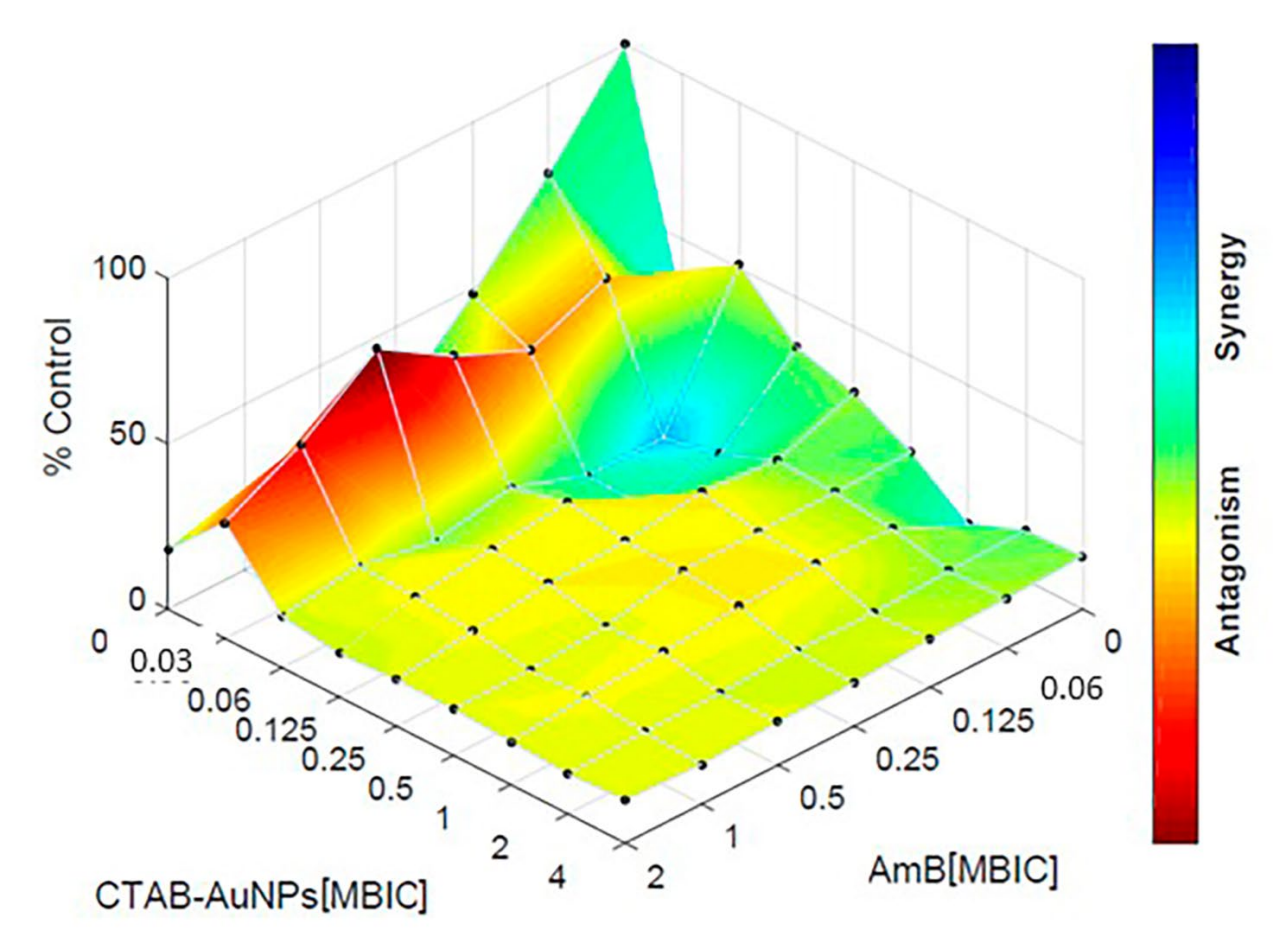
Combination dose-response based on the Bliss independence model. Interaction between cetyltrimethylammonium bromide gold nanoparticles (CTAB-AuNPs) and Amphotericin B (AmB) on 48 h-old mature biofilms, showing different interactions depending on the combination concentrations. FICi scores presented in the figures are the average of a minimum of two independent replicates

The checkerboard assay allowed us to evaluate the effect of the combination of each AuNP with AmB against C. *tropicalis* mature biofilms. The interpretation of the results by FICi values using MBIC endpoint parameters, as for the MIC at the planktonic level, is shown in Table 2. A biofilm reduction of 97.29% was obtained with CTAB-AuNPs plus AmB, corresponding to 0.05 FICi points (synergism). Meanwhile, by conjugating cys-AuNPs with AmB, a 91.66% biofilm reduction was obtained, resulting in a FICi value of 0.75 (additive) (Fig. 4). The interactions between AuNPs and AmB were interpreted by response surface analysis, based on the Bliss model, and revealed the mapping of the synergy levels on the experimental combination dose–response surface (Fig. 5). The microplate data were expressed as a percentage of growth compared to the growth control and subsequently transformed into a dose–response curve for each drug alone. The SUM-SYN-ANT metric exhibited a range from 5.69 to 36.06, with a mean value of 30.91. According to the experimental plate results, synergy was considered when the SUM-SYN-ANT was ≥ 20%, antagonism when ≤ − 20%, and indifference when the value fell between − 20% and 20%. A similar analysis to the checkerboard data (FICi) using the response surface approach yielded comparable results.

**Table 2:** Synergic antifungal activity of gold nanoparticles (AuNPs) with Amphotericin B (AmB) on planktonic and sessile cells of *Candida tropicalis*

	AmB	CTAB-AuNPs	FICi	INTPN	cys-AuNPs	FICi	INTPN
	**Planktonic cells**						
MIC	0.25	0.02	1	additive	0.04	1.50	indifferent
MIC combination	0.12	0.01			0.04		
	**Sessile cells**						
MBIC	200	0.31	0.5	synergism	0.63	0.75	additive
MBIC combination	50	0.07			0.31		

**Abbreviations**: Amphotericin B (AmB), cetyltrimethylammonium bromide gold nanoparticles (CTAB-AuNPs) and conjugated with cysteine (cys-AuNPs), minimum inhibitory concentration (MIC), minimum fungicidal concentration (MFC), minimal biofilm inhibitory concentration (MBIC), minimal biofilm eradication concentration (MBEC), interpretation (INTPN)

**Note**: All MIC and MFC data are expressed in terms of mM for AuNPs, and µg ml^− 1^ for AmB

**Fig. 6:**
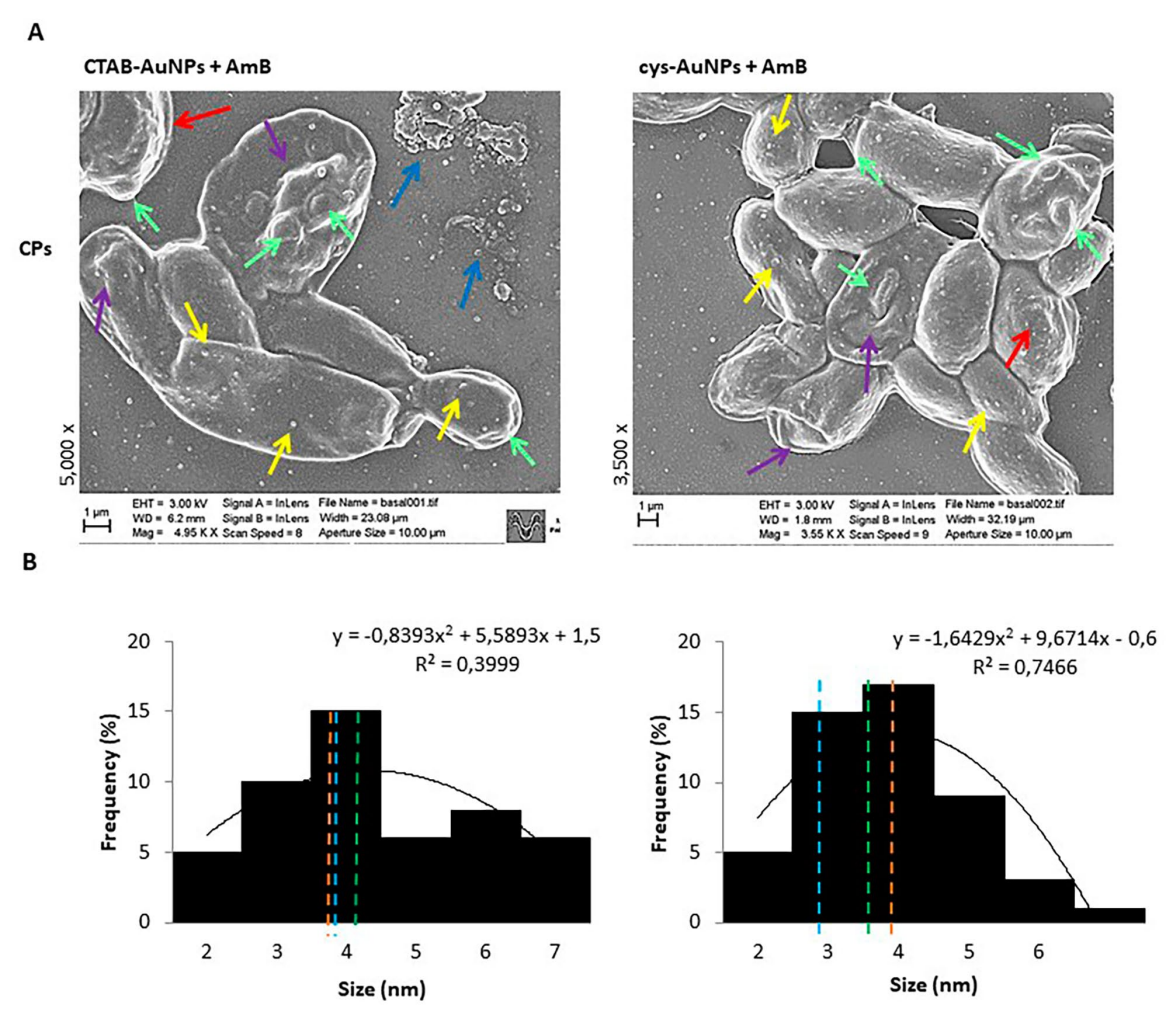
Scanning electron microscopy images of antifungal-tolerant persister cells (PCs) after combined treatment and size distribution histogram analysis. (**A**) SEM images (5,000x and 3,500x) showing the PCs after applying gold nanoparticles (AuNPs) plus Amphotericin B (AmB) combination and cell wall surface appearance. The AgNPs plus AmB caused important alterations in the size and shape of individual cells. The arrows show the AuNPs (yellow) on the cell wall, with aberrant morphology (purple), protuberance (green), and roughness (red) visible on the cell wall, and detritus cells with extracellular material leaking by lysis (light blue). Scale bar, 1 μm. (**B**) Histogram analysis showing the mean (green), median (orange), and mode (light blue) of the PC size distribution

By using SEM, the effect on PCs was observed after the joint effects of AuNPs plus AmB, with the number of cells being clearly seen to be reduced and of smaller sizes. Alterations were noted on the cell wall, no hyphae- or pseudohyphae-shaped cells were found, and the biofilm architecture was substantially eliminated (Fig. 6A). After CTAB-AuNPs plus AmB, aberrant morphology cells were observed, with protuberance(green) and roughness (purple) in the cell wall, and with the presence of detritus indicating cell damage caused by the loss of cellular integrity by lysis (light blue). After the joint effects of cys-AuNPsB plus AmB, the cell shape became only spheroid with small cells. In Fig. 6B, it can be observed how the size and shape of the PCs were affected. The average size of the yeast cells decreased (mean - green) to 4.37 ± 0.47 μm and 3.85 ± 0.14 μm for CTAB-AuNPs/AmB and cys-AuNPs/AmB, respectively, with the same D50 = 4 (orange) being found. However, the mode (the size most commonly found - light blue) was 4 and 3, respectively.

## Discussion

The search to find new antimicrobials for the prevention and treatment of *Candida* biofilm-associated infections has been gaining greater interest, with an increasing investigation into newer methods including novel nanotechnology-based approaches [16, 18, 40]. Although metal NPs have demonstrated a remarkable potential against bacterial infections, they have limited use in the treatment of fungal infections and have not been shown to be useful in new approaches to eradicate PCs within biofilms [19, 20, 41–43].

In the last decade, there has been a rise in the morbidity and mortality rates in *C. tropicalis*, with only a limited set of antifungals being available, and in some cases, the antifungal monotherapy lacks efficacy [1, 2]. The use of combination therapies is a well-known strategy in bacterial infections for improving efficacy and reducing toxicity. However, studies concerning the antifungal effect of metal NPs in combination with clinically used antifungal drugs are still scarce, despite the strategy of synergy between combinatory agents possibly aiding in the optimization of the current mycosis treatment and exerting less evolutionary pressure for fungal resistance [34, 35, 37, 44]. Lohse et al. combined different standard antifungal agents with protease inhibitors and obtained improvements in the eradication of *C. albicans* biofilms [34]. Previously, we demonstrated the synergistic activity of novel antifungal activity of oligostyrylbenzenes with low concentrations of AmB on planktonic cells and biofilms of *C. tropicalis* [23]. In this present investigation, we evaluated the potential antifungal combinations between AuNPs and the traditional antifungal drug AmB in an attempt to increase the number of potential targets for sessile and PC cells, improve the efficacy of yielding to synergy, and reduce AmB toxicity by decreasing the dosages, thereby helping to overcome the development of resistance.

PCs are a specialized case of tolerance and can only be detected by fungicidal drugs [45]. It is known that *Candida* PCs have not been detected in planktonic cultures as they appear to be specific to biofilms and have a range of variability between 0.012 and 2% that may be explained, at least in part, by the surface type of the formation of a biofilm [6, 32, 46]. Our results showed the presence of PCs in 48 h-old biofilms for CTAB-AuNPs and cys-AuNPs, with a characteristic biphasic killing pattern where the bulk of the susceptible sessile cells was rapidly exterminated, after which, a plateau appeared that was indicative of the population being enriched in surviving PCs. As the same values were obtained from the MIC determination of cells surviving the killing, this demonstrates that the survivors were not mutants. This fraction increased 4.1 and 4.9 times for CTAB-AuNPs and cys-AuNPs, respectively, compared to AmB-tolerant PCs, at the MBEC of each agent.

Ultrastructural analysis of the biofilm topography and architecture revealed that untreated biofilms displayed a uniform distribution with a tight clustering of cells which showed a characteristic dense network of EPS, hyphae, and yeast cells. In contrast, after being AuNP-treated, an important reduction in the mature biofilm was observed with less clustering (only microcolonies) and higher porosity. In addition, the size of the cells affected and the number of sessile cells were clearly reduced. Our results showed that, for all agents, no true hyphae or pseudohyphae-like shapes were present. This finding is important as both filamentation and the development of biofilms represent two of the main virulence factors of *Candida* species. A similar result has been recently reported by Vazquez-Munoz R et al., with silver NPs on *Candia auris* planktonic and biofilm growth phases being observed [47].

The objective of any agent’s combination is to achieve synergy through the joint mechanisms of action and to reduce the doses of traditional drugs. In our study, combination treatment with AuNPs and AmB-NPs not only significantly reduced the biofilm sessile cells, but also had a destructive effect on the architecture of the biofilm itself, thereby decreasing the number of PCs. Using SEM, we observed that the AuNPs adhered to the PC walls with a non-specific distribution, similar to previously reported studies [48, 49], but with different effects being found between the two nanoparticles. CTAB-AuNPs plus AmB activity induced aberrant morphologies on PCs and less clustering. However, after cys-AuNps plus AmB treatment, we observed that PCs remained attached to each other after cell division, leading to the formation of a few clustered cells, typically in groups of less than 10. These differences in behavior may be due to the different interactions between AmB and the AuNPs. By examining their chemical structures, we can infer how these interactions might occur. AmB exhibits a characteristic amphiphilic structure: its hydrophobic part is formed by a polyene chain of fifteen carbon atoms with alternating double and single bonds, and its main function is to interact with ergosterol, facilitating the formation of a pore in the membrane. The hydrophilic part is constituted by a polyol domain and two deoxysugars with a carboxylic acid and amine group. The polyol domain part is associated with the redox properties of AmB, the deoxysugars are referred to as “the polar head” due to the formation of a zwitterion [50]. On the other hand, CTAB-AuNPs are formed by a bilayer CTAB-coated gold-centered NP where the ammonium functional group interacts with the surface of the NP and towards the aqueous solution. This means that the CTAB-AuNPs present a strong positive charge, as indicated by the value of zeta potential of + 57.5 ± 4.3mV. Considering both structures described, AmB and CTAB-AuNP, the interaction between them is strongly electrostatic between the acid and hydroxyl groups of AmB and the ammonium groups of CTAB. In support of this, a study by Fotaki et al. demonstrates that AmB increases its solubility in water in the presence of CTAB [51]. Additionally, Manosroi et al. reported that AMB increased transdermal absorption with positively charged liposomes [52]. Both examples justify the interaction between AmB and CTAB-coated AuNPs. In the case of cys-AuNPs, the structure of the NP is different. In the synthesis, the sodium borohydride deprotonates the thiol, forming the thiolate which interacts with the AuNPs. For this reason, it shows a negative zeta potential of -40.93 ± 0.9 mV. The AuNPs interact with the thiolate group of cysteine, creating a monolayer of amino acids that orient the amino and carboxylic acid groups to the aqueous solution. At physiological pH, these amino acids are in their zwitterionic form as is AmB, which also has one amino and one carboxylic acid group in the polar head. This means that the protonated and positively charged amino groups interact with the negatively charged acidic groups of both structures in a double anchor. Pinheiro et al. demonstrated in their study that cysteine derivative increases the solubility of AmB in water, suggesting an interaction between the two [53].

Synergy was detected using a checkerboard, with the data being interpreted by two different approaches (FICi or response surface analysis). Combinations of CTAB-AuNPs plus AmB resulted in at least a 4-fold decrease in the MBIC values of mature *C. tropicalis* biofilm, thereby demonstrating synergy (FICi = 0.50), while the cys-AuNPs/AmB combination demonstrated an additive interaction (FICi = 0.75). In addressing the limitations of the FICi model, we employed an alternative approach based on the Bliss model, which is not dependent on an endpoint. Interestingly, this alternative model yielded consistent results, demonstrating the same interactions (synergy). A similar approach has been previously employed in testing drug interactions against yeasts [54]. Consistent with our results, Schwarz et al. also reported similar values of in vitro synergy, specifically for the combination of isavuconazole with colistin, between both methods for *Candida* species [35].

The mechanisms behind synergistic interactions are complicated. Studies combining cysteine and AmB have shown that cysteine inhibits its activity due to the antioxidant character of cysteine [19, 55]. This character is determined by the presence of the thiol group and its oxidation to disulfide bonds. Notably, in the case of cys-AuNP, this is not possible since the sulfur is bound to the AuNPs, which explains its synergistic effect with AmB, although we cannot rule out a mode of action of individual components that might help to explain the synergy observed in our results. In mature biofilms, the AuNPs, due to their size and large surface area to volume ratio, could diffuse easily through the biofilm matrix via the complex network of channels, voids, and pores. This allowed AuNPs to reach sessile cells easily in the inner regions of the biofilms and become attached and anchored to the surface of sessile cells by strong electrostatic interactions, as was revealed by SEM. AmB creates pores in the sessile cell membrane by forming aggregates with ergosterol. This can enhance AuNP uptake through membrane wall disruption and also act on intracellular targets, causing the antibiofilm effect to be maximized. It has been reported that Au ions can generate a broad spectrum of antimicrobial activity that inactivates cells by different mechanisms, including cellular stress. This can lead to damage to essential macromolecules such as polysaccharides, proteins, lipids, and DNA, and finally, cause cell death [48, 49]. We previously reported that CTAB-AuNPs of *C. tropicalis* produce an increase in reactive oxygen species (ROS), reactive nitrogen intermediates (RNI), and enzymatic and non-enzymatic antioxidant defenses. As a consequence, these affected the biofilm growth through the accumulation of ROS intra and extracellular and RNI. However, we previously reported that CTAB-AuNPs revealed a higher level of iROS, followed by the action of the superoxide dismutase (SOD), and reduced total glutathione (tGSH). As total antioxidant capacity levels were greater with CTAB-AuNPs, this indicates a differential stimulation in sessile cells [19], which could be implicated in the lower antifungal observed with additive interaction reported in this paper. At synergic values, the damage caused by CTAB-AuNPs on the biofilm markedly disturbed the topography and the three-dimensional network architecture, with these combined mechanisms of action creating pores that caused leakage of ions and other materials (AmB), leading to the alteration of vital cell functions. Then, the deeper AuNP penetration into the biofilms disrupted the biofilm matrix, and principally the generation of free radicals. This important mechanism of AuNPs may have led to damage to essential macromolecules and resulted in sessile and PC cell death.

## Conclusions

New insights into the action of AuNPs combined with first-line antifungal agents, such as AMB, against PCs may lead to an effective solution to *Candida* biofilm-associated infections. The combination of AuNPs and AmB increases antifungal activity as a result of their efficacy possibly being improved by combining different mechanisms of action. Thus, this combination is a promising strategy for improving efficacy, reducing dosages, shortening the duration of antifungal therapy, and consequently, reducing side effects. In addition, relapse may be prevented by the action of this combination on PCs directly associated with recurrent or chronic infections.
